# Death from Strangulation of a Portion of the Ileum

**Published:** 1870-01

**Authors:** Wm. L. Lincoln

**Affiliations:** Wabashaw, Minnesota


ARTICLE VIII. — *Death from Strangulation of a portion of the Ileum.* Reported by WM. L. LINCOLN, M.D., Wabashaw, Minnesota:

A SINGULAR case came under my observation, which, if it serve no other good, may throw its little share of light on the question lately brought again to my notice — How far can the lower bowels be filled by injection?

On the morning of November 8, 1868, about 10 o'clock, I was called to visit A. S., who was reported to be suffering from colic of a severe and obstinate form. The history of the case was this: He had eaten a hearty breakfast at his *camp* in the woods, where he was trapping, about 11 o'clock A.M., after which, for a couple of hours, he disposed of his game, and then he paddled his canoe and set traps until nightfall, after which he had walked to the town, some six miles. Between nine and ten in the evening he went with a friend in an oyster saloon, and ate a dish of raw oysters, and soon after retired for the night. About three o'clock A.M. he awoke with severe pain in the umbilical region, and suffering some nausea. He drank warm water, with mustard, from which he obtained free emesis without any relief of pain. He also applied mustard paste to his bowels. Afterwards he took some remedies prescribed by a Homœopathist who was at hand, and continued to grow worse until his sufferings were past enduring. His countenance portrayed extreme agony, and his first words, on my entering the room, were: "O, doctor, can't you do something for me quick? If you can't, I shall die!" He located his pain in the umbilical region. There was no tenderness on pressure over any part of the abdomen; no coat on his tongue, and his pulse was but slightly accelerated. The symptoms pointed to intussusception.

He got, by endermic injection, one-fourth grain morph. sulph., and directing warm water to be had in readiness, I left him. Returning in fifteen minutes, found him perfectly free from pain. I then proceeded to prepare a rectum tube by winding a wet rag around it to form a collar to prevent escape of the fluid, and proceeded to inject six quarts of water, not leaving half a teacupfull in the vessel, when he desired a passage, and evacuated nearly the same quantity as injected. About five hours after the endermic injection, the pain returned, not less violent than at first; but a repetition of the injection was followed by the same happy effect. In the succeeding twenty-four hours, I repeated the injection per rectum, twice, each time using about the same quantity as at first, with no evacuation except the water injected. It was necessary to repeat the endermic injection every five or six hours, for when the opiate effect was past, his shrieks would disturb the neighborhood. In the evening of Wednesday, the 11th, he had stercoracious vomiting, and sank rapidly for some hours, but rallied on the morning of Thursday, and pain returued not less severe than formerly. The injections of morphia gave prompt and full relief, and were repeated every five or six hours, until Sunday, rhe 15th, in the afternoon, when he vomited a fluid having a fœcal odor, which was followed, in a few hours, with death.

Monday, 16th, eighteen hours after death, I made a *post-mortem* examination, assisted by Dr. Milligan, who I invited to be present.

On making the abdominal section, there was no appearance of serous inflammation on intestines or abdominal walls; but the cause of the pain and death was at once revealed. A knuckle of the ileum, some five or six inches from the ileo-cœcal juncture, was tied in a complete knot; about seven inches of the ileum having passed through the knot, and being ligated thereby. The mucous membrane of that part of the intestine forming the knot, was thickened and inflamed, and highly vascular. The knuckle presented less departure from a normal condition. That no one may be in doubt as to the kind of knot formed in the ileum, will instruct how to form the knot: Take a rope or string, and make fast the two ends to some object; now take the free end, which is a knuckle, and pass a single knot in it, as near the end as you can. You will at once perceive that, if to your rope you had attached a piece of cloth to represent the mesentery, you could tie the same knot. I make this remark, because some physicians to whom a relative of my patient narrated the case, declared that such a knot was an impossibility.

The case proves to my mind that nature can do what the surgeon can neither do nor undo, and that water can be injected into the rectum so as to fill the colon to the ileo-cœcal valve.

